# Inhibition of the JAK2/STAT3 pathway in ovarian cancer results in the loss of cancer stem cell-like characteristics and a reduced tumor burden

**DOI:** 10.1186/1471-2407-14-317

**Published:** 2014-05-06

**Authors:** Khalid Abubaker, Rodney B Luwor, Hongjian Zhu, Orla McNally, Michael A Quinn, Christopher J Burns, Erik W Thompson, Jock K Findlay, Nuzhat Ahmed

**Affiliations:** 1 Women’s Cancer Research Centre, Royal Women’s Hospital, 20 Flemington Road, Parkville, Melbourne, Victoria 3052, Australia; 2 Department of Surgery, St Vincent’s Hospital, University of Melbourne, Melbourne, Victoria 3065, Australia; 3 Department of Surgery, University of Melbourne, Royal Melbourne Hospital, Melbourne, Victoria 3052, Australia; 4 Department of Obstetrics and Gynaecology, University of Melbourne, Melbourne, Victoria 3052, Australia; 5 Walter and Eliza Hall Institute of Medical Research, Melbourne, Victoria 3052, Australia; 6 St Vincent’s Institute, Melbourne, Victoria 3065, Australia; 7 Prince Henry’s Institute of Medical Research, Melbourne, Victoria 3168, Australia

**Keywords:** Ovarian carcinoma, Cancer stem cell, Metastasis, Ascites, Chemoresistance, Recurrence, JAK2/STAT3 pathway

## Abstract

**Background:**

Current treatment of ovarian cancer patients with chemotherapy leaves behind a residual tumor which results in recurrent ovarian cancer within a short time frame. We have previously demonstrated that a single short-term treatment of ovarian cancer cells with chemotherapy *in vitro* resulted in a cancer stem cell (CSC)-like enriched residual population which generated significantly greater tumor burden compared to the tumor burden generated by control untreated cells. In this report we looked at the mechanisms of the enrichment of CSC-like residual cells in response to paclitaxel treatment.

**Methods:**

The mechanism of survival of paclitaxel-treated residual cells at a growth inhibitory concentration of 50% (GI50) was determined on isolated tumor cells from the ascites of recurrent ovarian cancer patients and HEY ovarian cancer cell line by *in vitro* assays and in a mouse xenograft model.

**Results:**

Treatment of isolated tumor cells from the ascites of ovarian cancer patients and HEY ovarian cancer cell line with paclitaxel resulted in a CSC-like residual population which coincided with the activation of Janus activated kinase 2 (JAK2) and signal transducer and activation of transcription 3 (STAT3) pathway in paclitaxel surviving cells. Both paclitaxel-induced JAK2/STAT3 activation and CSC-like characteristics were inhibited by a low dose JAK2-specific small molecule inhibitor CYT387 (1 μM) *in vitro*. Subsequent, *in vivo* transplantation of paclitaxel and CYT387-treated HEY cells in mice resulted in a significantly reduced tumor burden compared to that seen with paclitaxel only-treated transplanted cells. *In vitro* analysis of tumor xenografts at protein and mRNA levels demonstrated a loss of CSC-like markers and CA125 expression in paclitaxel and CYT387-treated cell-derived xenografts, compared to paclitaxel only-treated cell-derived xenografts. These results were consistent with significantly reduced activation of JAK2 and STAT3 in paclitaxel and CYT387-treated cell-derived xenografts compared to paclitaxel only-treated cell derived xenografts.

**Conclusions:**

This proof of principle study demonstrates that inhibition of the JAK2/STAT3 pathway by the addition of CYT387 suppresses the ‘stemness’ profile in chemotherapy-treated residual cells *in vitro*, which is replicated *in vivo,* leading to a reduced tumor burden. These findings have important implications for ovarian cancer patients who are treated with taxane and/or platinum-based therapies.

## Background

Current treatment for advanced-stage ovarian cancer patients consists of aggressive surgery followed by chemotherapy to eradicate the residual disease [1,2]. Postoperatively, all women, except those diagnosed with Stage 1 well differentiated tumors are given platinum (cisplatin or carboplatin) and taxane (paclitaxel)-based chemotherapies, resulting in initial remission in up to 80% of patients. Unfortunately, the majority of these patients relapse within two years, resulting in a 5-year survival rate of only 27% [3]. This low survival rate is largely due to the presence of chemotherapy-resistant residual tumor cells which have the capacity to withstand the cytotoxic effects of therapies and repopulate, leading to recurrence [4]. Previous studies on the mechanisms underlying the failure of taxane and cisplatin-based chemotherapy have implicated enhanced expression of multidrug transporters [5], involvement of anti-apoptotic pathways [6], mutations in the p53 pathway [7,8], increased glutathione and metallothionein levels [9], altered expression of tubulin binding proteins [10], expression of taxane metabolizing proteins, altered cell signaling resulting in reduced apoptosis [11] and epithelial mesenchymal transition (EMT) [12-14].

Ovarian cancer is a disease commonly complicated by the presence of ascites in the abdominal cavity [3,15]. As the disease progresses tumor cells are shed in the ascites by the rupture of the primary tumor surface [2]. Aggregates of tumor cells commonly known as ‘spheroids’ float freely in an anchorage independent condition in ascites [16-19]. This transceolomic route of ovarian cancer metastasis has been suggested due to the development of ovarian cells from the coelomic mesothelium during embryogenesis [20]. The attachment of spheroids to the peritoneum has been shown to be facilitated by cell surface proteins such as CD44, collagen 1 and β1 integrin which facilitate adhesion to the mesothelial cells lining the peritoneal cavity [21,22]. Once attached to the peritoneal surface, cancer cells proliferate and invade the mesothelium (outer layer of the peritoneal membrane) [23]. It is thought that this process of seeding of the peritoneum is directly associated with the production of ascites, evidenced by the reduction of ascites volume when patients undergo debulking surgery or chemotherapy treatment that removes the majority of residual macroscopic disease [3,15]. Along with transcoelomic metastatic tumors, extensive seeding of cancer cells on various abdominal organs such as the colon, uterus and omentum is commonly observed in the late-stage disease [2].

The presence of cancer stem cells (CSCs) in the ascites of ovarian cancer patients was demonstrated nearly eight years ago [24]. In recent studies, the presence of CSCs in ovarian cancer has been shown by using side population sorting or by sorting cells using specific cell surface markers and intracellular expression of proteins (CD44, My88, CD133, CD117, CD24, ALDH1) commonly considered to be CSC markers [25-30]. CSCs have been demonstrated to produce greater tumor burden and to be resistant to chemotherapy [31,32]. In recent studies we and others have shown recurrent ovarian tumors to be enriched with CSCs and mediators of pathways that regulate CSCs, suggesting that CSCs may contribute to the development of recurrence [33,34].

The JAK2/STAT3 pathway mediates the effects of many growth factors and cytokines by regulating the expression of downstream gene expression [35]. In normal cells, the JAK2/STAT3 pathway is transiently activated in response to specific growth factors and cytokines (IL6, GCSF, LIF, EGF, etc.). However, in cancer cells, including breast, ovarian and prostate, the JAK2/STAT3 pathway is constitutively active in the majority of cases [36,37]. We and others have previously shown nuclear localization of activated phosphorylated STAT3 in more than 70% of high-grade serous ovarian cancer, where it was associated with decreased survival [36,38]. This pathway has been linked with cancer cell survival and chemoresistance in ovarian, as well as number of other solid cancers [13,39,40].

CYT387 is a specific JAK2 inhibitor which is in clinical development as treatment for a diverse range of diseases, including myelofibrosis [41] and myeloma [42]. CYT387 demonstrated efficacy in a *JAK2*V617F mutation-associated animal model where it inhibited constitutively activated JAK2 associated STAT3 function by neutralizing IL-6 by a negative feed-back inhibition [41]. The compound showed a negligible effect on the metabolism of other agents and is unlikely to participate in metabolic drug-drug interactions [41]. Preclinical analysis has shown that CYT387 was well tolerated when administered to mice orally at doses up to 50 mg/kg of body weight, with no sign of overt toxicity [41].

In this study, we demonstrate that a short-term single exposure of CYT387 in addition to paclitaxel reduces the CSC-like characteristics and activation of JAK2/STAT3 pathway promoted by paclitaxel in residual cells *in vitro*. The *in vitro* suppression of CSC-like characteristics and activation of JAK2/STAT3 pathway by CYT387 is mimicked in *in vivo* mouse xenografts with a reduced tumor burden. These data emphasize the need to explore further the effect of CYT387 in combination with chemotherapy in pre-clinical ovarian cancer models.

## Methods

### Cell line

The human ovarian HEY cell line was derived from a peritoneal deposit of a patient diagnosed with papillary cystadenocarcinoma of the ovary [43]. The cell line was grown as described previously [44].

### Antibodies and reagents

Polyclonal antibody against phosphorylated (Tyr-705) STAT3 (P-STAT3), total STAT3 (T-STAT3), phosphorylated (Tyr-1007/1008) JAK2 (P-JAK2), total JAK2 (T-JAK2) and GAPDH were obtained from Cell Signalling Technology (Beverly, MA, USA). Antibodies against cytokeratin 7 (cyt7), Ki67, CA125, E-cadherin, vimentin, Oct4 and CD117 (c-Kit) used for immunohistochemistry were obtained from Ventana (Roche, Arizona, USA). CYT387 was obtained from Gilead Sciences (CA, USA).

### Patients

#### ***Human ethics statement***

Ascites was collected from patients diagnosed with Stages IIa-IV serous ovarian carcinoma and adenocarcinoma Not Otherwise Specified (NOS) (Table 1), after obtaining written informed consent under protocols approved by the Human Research and Ethics Committee (HREC approval # 09/09) of The Royal Women’s Hospital, Melbourne, Australia. HREC approval #09/09 also obtained consent from participants to publish the results from this study provided anonymity of patients is maintained at all times.

**Table 1:** Description of the patients recruited for the study

**Samples**	**Stage**	**Grade**	**Treatment cycles**	**Age**	**Overall survival**
Ascites 1	IIIc	High Grade Serous	Carboplatin and Paclitaxel 6 cycle	39 years at diagnosis	3 years and 7 months
			Doxorubicin Pegylated Liposomal 9 cycles		
			Gemcitabine and Carboplatin 3 cycles		
			Paclitaxel (12 treatments in cycle 1, 3 treatments in cycles 3 through to 9)		
Ascites 2	IIa	High Grade Serous	Carboplatin 5 cycles	78 years at diagnosis	6 months
Ascites 3	Unknown	Not Graded	Carboplatin and Paclitaxel 4 cycles	59 years at diagnosis	5 months as of 20/11/2012
Ascites 4	Unknown	Adenocarcinoma NOS	Carboplatin and Paclitaxel 6 cycles	75 years at diagnosis	1 year 8 months
			Tamoxifen 2 cycles		
			Doxorubicin Pegylated Liposomal 4 cycles		
Ascites 5	IIc	High Grade Serous	Carboplatin and Paclitaxel 4 cycles, Topotecan 1 cycle	64 years at diagnosis	5 months
Ascites 6	IIIc	High Grade Serous	Carboplatin and Paclitaxel 6 cycles	52 years at diagnosis	2 years 5 months
			AMG-386 182 Trial 8 cycles		
			Paclitaxel 3 cycles		
			Paragon Trial 2 cycles		
			Carboplatin single agent 3 cycles		
			Cyclophosphamide 2 cycles		
Ascites 7	IIIc	High Grade Serous	Carboplatin and Paclitaxel 9 cycles	59 years at diagnosis	2 years 6 months
			Cisplatin 4 cycles		
			Cyclophosphamide 2 cycles		
Ascites 8	IV	Adenocarcinoma NOS	Carboplatin and Paclitaxel 6 cycles	67 years at diagnosis	2 years 6 months
			Gemcitabine and Carboplatin 6 cycles		
Ascites 9	Unknown	Adenocarcinoma NOS	Cyclophosphamide 3 cycles		2 years 8 months
			Carboplatin and Paclitaxel 6 cycles	65 years at diagnosis	
			MORAb Trial 9 cycles		
			Doxorubicin Pegylated Liposomal 3 cycles		
Ascites 10	IIIc	High Grade Serous	Doxorubicin Pegylated Liposomal 3 cycles	55 years	5 years 5 months
			ICON 7 Trial 18 cycles		
			ICON 6 Trial 6 cycles		
			Paragon Trial 1 cycle		
			Paclitaxel 6 cycles		
Ascites 11	IIIc	High Grade Serous	Hormonal Therapy Tamoxifen	69 years at diagnosis	7 years 11 months
			Topotecan Hydrochloride 2 cycles		
			Carboplatin and Paclitaxel 6 cycles		
			Carboplatin single agent 6 cycles		
			Gemcitabine and Carboplatin 6 cycles		
			Carboplatin single agent 6 cycles		
			Cyclophosphamide 6 cycles		
			Doxorubicin Pegylated Liposomal 4 cycles		
			Paclitaxel 3 cycles		
Ascites 12	IIIc	High Grade Serous	Doxorubicin Pegylated Liposomal 3 cycles Carboplatin and Paclitaxel 6 cycles	59 years at diagnosis	2 years 11 months as of 21/05/2013
			Gemcitabine and Carboplatin 6 cycles		
Ascites 13	IIIc	High Grade Serous	Doxorubicin Pegylated Liposomal 4 cycles	53 years at diagnosis	2 years 11 months as of 21/05/2013
			Carboplatin and Paclitaxel 6 cycles		
			AMG-386 182 9 cycles		
			Paclitaxel 6 cycles		
			Cyclophosphamide 2 cycles		
			Topotecan 2 cycles		
Ascites 14	IV	High Grade Serous	Carboplatin 1 cycle	46 years at diagnosis	2 years 6 months as of 13/08/2013
			Carboplatin and Paclitaxel 6 cycles		
Ascites 15	IIIc	Not Graded	Carboplatin 5 cycles	76 years at diagnosis	1 year and 8 months
			Cyclophosphamide 7 cycles		
			Paragon Trial 3 cycles		

*NOS*, Not Otherwise Specified.

The histopathological diagnosis, including tumor grades and stage was determined by independent staff pathologists as part of the clinical diagnosis (Table 1). Ascites was collected as they were received by the laboratory. For collection of ascites preference was given to samples obtained from patients diagnosed with serous ovarian cancer. However, to meet the experimental demand samples from three patients diagnosed with adenocarcinomas NOS were also included. Ascites was collected from patients at the time of recurrence. Patients in this group were not all treated identically and had previously received combinations of chemotherapy consisting of paclitaxel, carboplatin and other drugs such as doxorubicin, gemcitabine, docetaxel, cyclophosphamide and topotecan after each recurrent episode (Table 1).

### Preparation of tumor cells from ascites of ovarian cancer patients

Tumor cells from ascites were isolated as described previously [34]. Briefly, 500 ml of ascites was used to collect tumor cells. The ascites cells were seeded on low attachment plates (Corning Incorporated, NY) in MCDB:DMEM (50:50) growth medium supplemented with fetal bovine serum (10%), glutamine (2 mM) and penicillin/streptomycin (2 mM) (Life Technologies, CA, USA) after removal of the red blood cells by hypotonic shock using sterile MilliQ water as described previously [34]. Cells were maintained at 37°C in the presence of 5% CO_2_ and tumor cells floating as non-adherent population were collected after 2–3 days, and screened for CA125, EpCAM, cytokeratin 7 (CK7) and fibroblast surface protein (FSP) by Flow Cytometry to assess their purity. Cells were passaged weekly and experiments were performed within 1–2 passages.

### Treatment of HEY and isolated tumor cells with paclitaxel, CYT387 or combination of both

Isolated ascites tumor cells and ovarian cancer cell line HEY were treated with paclitaxel concentrations at which 50% growth inhibition was obtained (GI50 ~ 6 ng/ml for ascites tumor cells and 1 ng/ml for HEY cells for three days) [45]. For CYT387 treatment, cells were screened for the response to different concentrations of CYT387 in HEY cells. The concentration of CYT387 that gave optimum inhibition of the active (phosphorylated) JAK2/STAT3 pathway by Western blot in response to paclitaxel in HEY cells was ~1 μM, and as such, 1 μM CYT387 was used throughout the study. For combination treatment, ascites-derived tumor cells were treated with 6 ng/ml of paclitaxel and 1 μM of CYT387, while the HEY cells were treated with 1 ng/ml of paclitaxel and 1 μM of CYT387. Treatment was performed for three days.

### Immunofluorescence analysis

Immunofluorescence analysis of β-tubulin, ERCC1, EPCAM, CD117, NANOG, Oct-4, P-STAT3, T-STAT3, P-JAK2 and T-JAK2 was performed as described previously [34]. Images were captured by the photo multiplier tube (PMT) using the Leica TCS SP2 laser, and viewed on a HP workstation using the Leica microsystems TCS SP2 software. The mean fluorescence intensity was quantified using Cell-R software (Olympus Soft Imaging Solution). When calculating mean fluorescence intensities a comparative field of view with equal number of cells was chosen for each analysis to compensate for the disparity between cell numbers in the wells containing treated and untreated cells. As such, the calculations were performed on equal number of cells.

### RNA extraction and Real Time-PCR (q-PCR)

Solid tumors derived from mice inoculated with HEY cells were homogenised using PowerLyzer™ 24 (MO BIO Laboratories Inc, Carlsbad CA, United States) according to manufacturer’s instruction. RNA was extracted from the homogenised xenograft and cDNA synthesised as described previously [34]. Quantitative determination of mRNA levels of various genes was performed in triplicate using SYBR green (Applied Biosystems, Australia) as described previously [34]. The primers for Oct-4, NANOG, CD44, CD117, and EpCAM have been described previously [14].

### SDS-PAGE and Western blot analysis

SDS-PAGE and Western blot was performed on cell lysates by the methods described previously [14]. Protein loading was monitored by stripping the membrane with Restore Western blot Stripping Buffer (Thermo Scientific, MA, USA) and re-probing the membrane with β-actin primary antibody (Sigma-Aldrich, Sydney, Australia).

### ^3^[H]-Thymidine assay

^3^[H]-Thymidine uptake assay as a measure of cell proliferation was performed as described previously [34]. Briefly, 1×10^5^ HEY cells or ascites-derived tumor cells untreated or treated with paclitaxel or CYT387 + paclitaxel were seeded in triplicate on 24 well plates. After 3 days, 0.5 μCi of [^3^H] thymidine was added to each well, and cells were incubated at 37°C for an additional 16 h. Cells were washed with PBS, harvested and lysed in 1% Triton and incorporation of [^3^H] thymidine was measured by liquid scintillation counting (Hidex 300SL, LKB Instruments, Australia).

### Animal studies

#### ***Animal ethics statement***

This study was carried out in strict accordance with the recommendations in the Guide for the Care and Use of the Laboratory Animals of the National Health and Medical Research Council of Australia. The experimental protocol was approved by the Ludwig Institute/Department of Surgery, Royal Melbourne Hospital and University of Melbourne’s Animal Ethics Committee (Project-006/11), and was endorsed by the Research and Ethics Committee of Royal Women’s Hospital Melbourne, Australia.

### Animal experiments

The animal experiments were performed as described previously [45]. Briefly, female Balb/c *nu/nu* mice (age, 6–8 weeks) were obtained from the Animal Resources Centre, Western Australia. Animals were housed in a standard pathogen-free environment with access to food and water. HEY cells were treated with paclitaxel (1 ng/ml) or CYT387 (1 μM) or paclitaxel (1 ng/ml) plus CYT387 (1 μM) as described previously. 5×10^6^ cells surviving treatments after three days were injected intraperitoneally (ip) in nude mice. Mice were inspected weekly and tumor progression was monitored based on overall health and body weight until one of the pre-determined endpoints was reached. Endpoint criteria included loss of body weight exceeding 20% of initial body weight and general pattern of diminished well-being such as reduced movement and lethargy resulting from lack of interest in daily activities. Mice were euthanized and organs (liver, stomach, lungs, gastrointestinal tract, pancreas, uterus, skeletal muscle, colon, kidney, peritoneum, ovaries and spleen) and solid tumors were collected for further examination. Metastatic development was documented by a Royal Women’s Hospital pathologist according to histological examination (H & E staining) of the organs.

### Immunohistochemistry of mouse tumors

For immunohistochemistry, formalin fixed, paraffin embedded 4 μm sections of the xenografts were stained using a Ventana Benchmark Immunostainer (Ventana Medical Systems, Inc, Arizona, USA) previously [45]. Immunohistochemistry images were taken using Axioskop 2 microscope, captured using a Nikon DXM1200C digital camera and processed using NIS-Elements F3.0 software. Images were scored independently by four reviewers blind to the molecular data as previously described [46].

### Statistical analysis

Data are presented as mean ± SEM. Treatment groups were compared with the control group using one way- ANOVA and Dunnett’s Multiple Comparison post-tests. A probability level of p < 0.05 was adopted throughout to determine statistical significance.

## Results

### Treatment of isolated tumor cells with paclitaxel resulted in the enhanced expression of ERCC1 and β-tubulin-III

**Figure 1:**
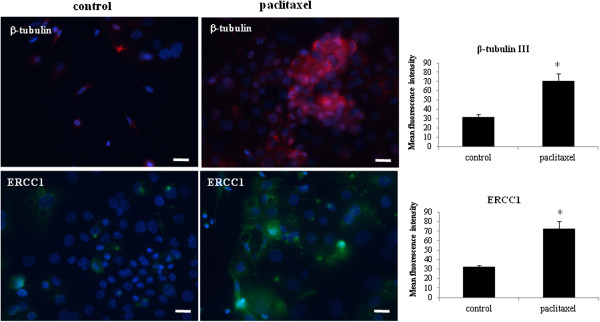
**Increased expression of β-tubulin III and ERCC1 in ascites-derived tumor cells in response to paclitaxel.** Expression and immunolocalisation of β-tubulin III and ERCC1 in ascites-derived tumor cells was evaluated by immunofluorescence using mouse monoclonal (green) and rabbit polyclonal (red) antibodies as described in the Methods. Cellular staining was visualized using secondary Alexa 488 (green) and Alexa 590 (red) fluorescent labelled antibodies while nuclear staining was visualized using DAPI (blue) staining. Images are representative of three independent experiments from three independent patient samples. The mean fluorescence intensity of β-tubulin III and ERCC1 was quantified using Cell-R software. Significant variations between the groups are indicated by *P < 0.05. Magnification 200×; scale bar = 10 μM.

Tumor cells from ascites were isolated as described previously [34]. The expression of ERCC1 and β-tubulin III were analysed by immunofluorescence staining in isolated tumor cells from ascites (control) and its paclitaxel-treated (6 ng/ml for 3 days) counterpart. In three ascites samples (Ascites 1–3, Table 1), very few control cells displayed ERCC1 staining which was confined mainly within the nuclear envelope (Figure 1). Cells from the same ascites samples treated with paclitaxel demonstrated a significantly higher number of ERCC1 stained cells and the scattered staining was seen in the nucleus as well as the cytoplasm (Figure 1). A similar enhancement in staining was observed for β-tubulin III, with paclitaxel surviving cells showing significantly enhanced staining when compared to their matched control cells (Figure 1). Quantitative measurement of three independent patient samples demonstrated a significant enhancement of β-tubulin III and ERCC1 staining in cancer cells surviving paclitaxel treatment *in vitro,* compared to their matched control counterparts (Figure 1).

### Paclitaxel treatment enhanced the expression of CSC markers in ascites-derived isolated tumor cells

**Figure 2:**
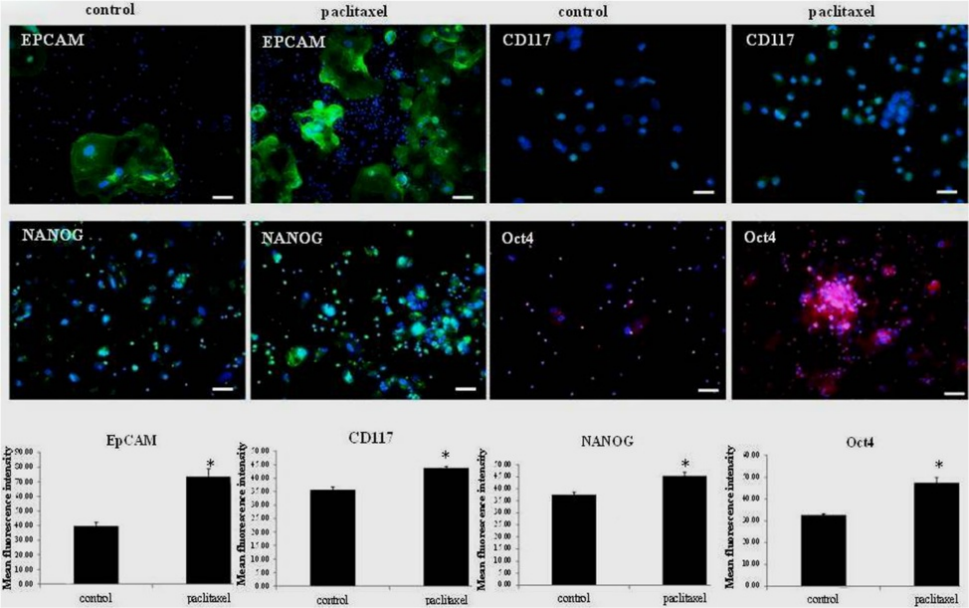
**Increased expressions of CSC and embryonic stem cell markers in ascites- derived tumor cells in response to paclitaxel.** Expression and localisation of EpCAM, CD117, Oct4 and NANOG in ascites-derived tumor cells in response to paclitaxel treatment was evaluated by immunofluorescence as described in Figure 1. Images are representative of three independent experiments from three independent patient ascites samples. The mean fluorescence intensity of CSC markers CD117, EpCAM and the embryonic stem cell markers NANOG and Oct4 expression in ascites-derived tumor cells was quantified using Cell-R software. Significant variations between the groups are indicated by *P < 0.05. Magnification 200×; scale bar = 10 μM.

Isolated tumor cells from the ascites of recurrent ovarian cancer patients (Ascites 3–5, Table 1) were subjected to paclitaxel treatment *in vitro* (6 ng/ml over three days). After three days of treatment, paclitaxel surviving tumor cells were analysed for the expression of CSC markers using immunofluorescence and compared with their control untreated counterparts (Figure 2). Staining of EpCAM and CD117 were confined mostly to cell membrane, while the staining of embryonic stem cell markers NANOG and Oct4 were localised both in the cytoplasm and nucleus (Figure 2). With paclitaxel treatment greater nuclear staining of NANOG and Oct4 were observed compared to control untreated cells (Figure 2). Quantitative measurements of CSC markers examined by immunofluorescence imaging revealed a significant enhanced staining of CSC markers EpCAM, CD117 and the embryonic stem cell markers Oct4 and NANOG, suggesting that the paclitaxel surviving population were enriched for CSC-like markers (Figure 2).

In order to determine if the expression of CSCs as deduced by immunofluorescence was consistent at mRNA level q-PCR was performed on isolated ascites cells treated with and without paclitaxel (Ascites 4, 5, 7 and 9, Table 1) (Additional file 1: Figure S1). The expression of CD117, Oct4 and JAGGED was significantly up in paclitaxel-treated ascites tumor cells, while there was a trend in the increased expression of EpCAM, CD44 and NANOG but it was not significant compared to untreated control.

### Paclitaxel treatment activated the JAK2/STAT3 pathway in ascites-derived tumor cells

**Figure 3:**
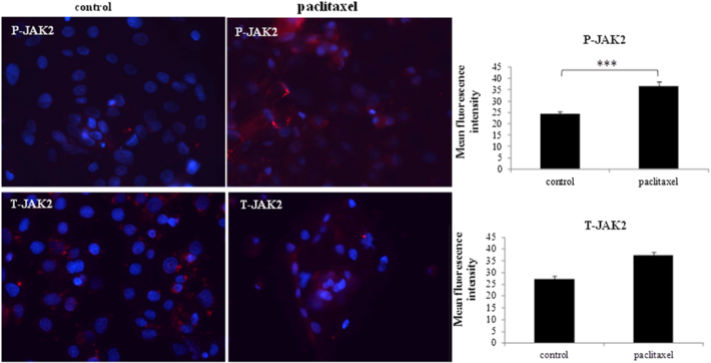
**Expression and localisation of P-JAK2 and T-JAK2 in ascites-derived tumor cells in response to paclitaxel treatment.** The images were evaluated as described in Figure 1. Images are representative of four independent experiments from four patient samples. The mean fluorescence intensity of P-JAK2 and T-JAK2 was quantified using Cell-R software. Significant intergroup variations are indicated by ***P < 0.001. Magnification 200×; scale bar = 10 μM.

**Figure 4:**
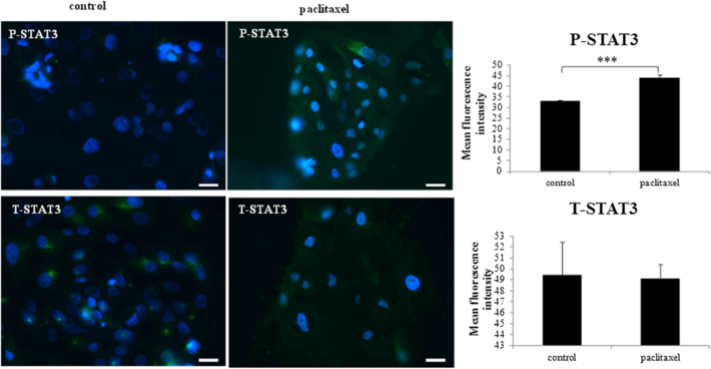
**Expression and localisation of P-STAT3 and T-STAT3 in ascites-derived tumor cells in response to paclitaxel treatment.** The images were evaluated as described in Figure 1. Images are representative of four independent experiments from four patient samples. The mean fluorescence intensity of P-STAT3 and T-STAT3 was quantified using Cell-R software. Significant intergroup variations are indicated by ***P < 0.001. Magnification 200×; scale bar = 10 μM.

Isolated ascites-derived tumor cells from four patients (Ascites 5, 6, 7 and 8 Table 1) were treated with paclitaxel and the activation of JAK2 (Tyr1007/1008) and STAT3 (Tyr-705) were analysed by immunofluorescence. Paclitaxel treatment resulted in the significant phosphorylation of JAK2 (P-JAK2) (Figure 3) and downstream STAT3 (P-STAT3) (Figure 4) in paclitaxel surviving cells, compared to their matched control counterparts. The expression of P-JAK2 in treated cells was mainly membrane bound and cytoplasmic. The expression of P-STAT3 was seen both in nucleus and cytoplasm of the treated cells. In all ascites samples tested, no significant difference in the level of total JAK2 (T-JAK2) and STAT3 (T-STAT3) between the control and paclitaxel surviving cells could be deduced by immunofluorescence (Figures 3 and 4).

### Paclitaxel treatment activated the JAK2/STAT3 pathway in chemotherapy surviving HEY cells; CYT387 inhibited paclitaxel-induced JAK2/STAT3 activation

**Figure 5:**
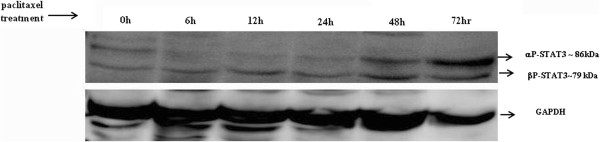
**Activation of STAT3 in response to paclitaxel treatment in HEY cells.** HEY cells were treated with paclitaxel (1 ng/ml) for 6, 12, 24, 48 and 72 hours. Cell lysates were prepared and Western blot was performed as described in the Methods. Total protein loading was determined by probing the membranes for GAPDH. Results are representative of three independent experiments.

**Figure 6:**
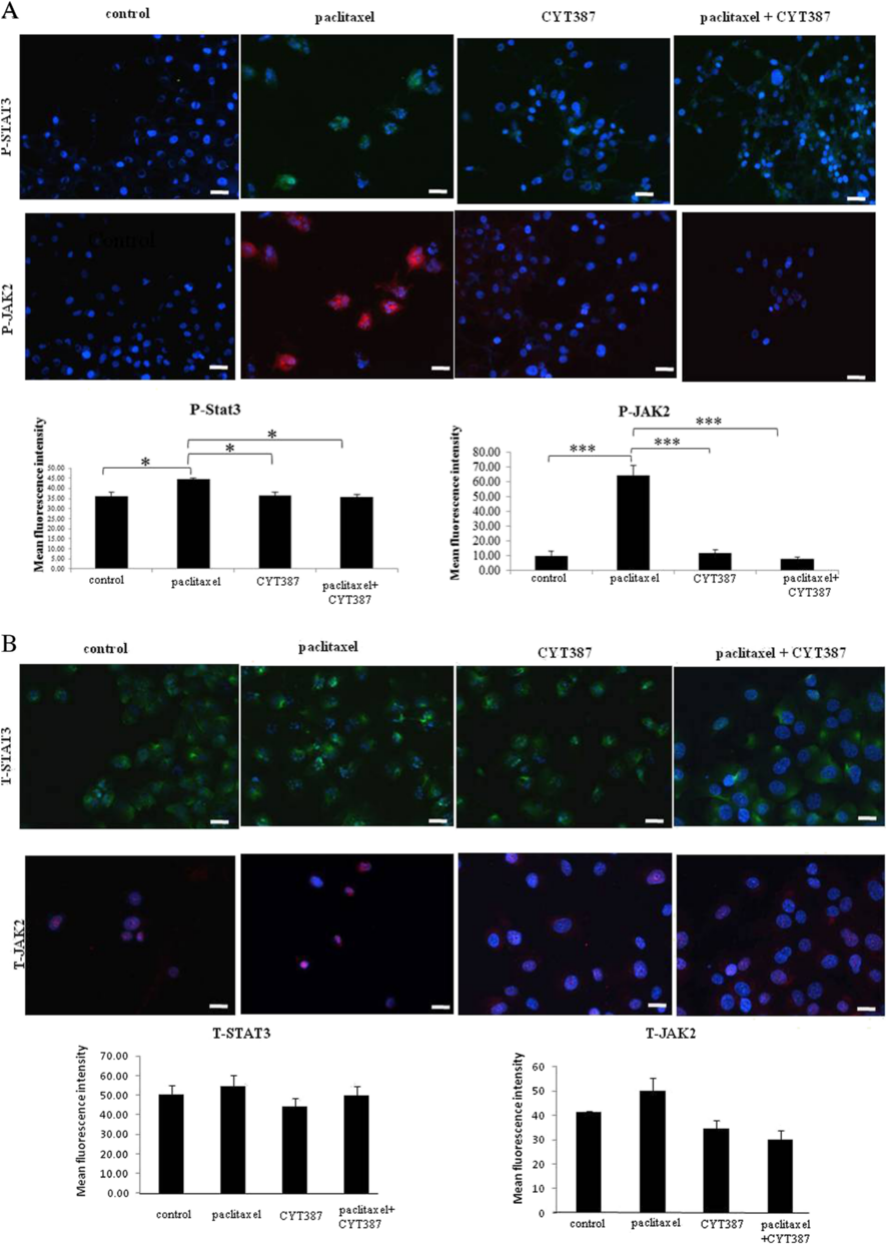
**Expression of phospho and total JAK2 and STAT3 in control, paclitaxel and paclitaxel plus CYT387-treated HEY cells. (A)** Expression and immunolocalisation of phospho (P)-JAK2 (Tyr-1007/1008) and phospho (P)-STAT3 (Tyr-705) in control, paclitaxel, CYT387 and combination of both treatments in HEY cell line was evaluated by immunofluorescence. Three independent experiments were performed in triplicate. The mean fluorescence intensity was quantified using Cell-R software. Significant variations between the groups are indicated by *P<0.05, *** P < 0.001. **(B)** The expression of total (T)-JAK2 and total (T)-STAT3 was evaluated and quantified as described in Figure 6**A**. Magnification 200x; scale bar = 10 μM.

Consistent with the ascites-derived tumor cells, treatment with paclitaxel resulted in the activation of the JAK2/STAT3 pathway in the ovarian cancer HEY cell line, resulting in a marked increase of both phosphorylated αSTAT3 (~86 kDa) and βSTAT3 (79 kDa) at two and three days post-treatment by Western blot (Figure 5). This observation was confirmed by immunofluorescence which demonstrated significant enhancement in the level of phosphorylated JAK2 (Tyr-1007/1008) and downstream STAT3 (Tyr-705) compared to control untreated cells (Figure 6A). Both P-JAK2 and P-STAT3 in paclitaxel-treated cells were found to be localised in the nucleus as well as cytoplasm of the paclitaxel-treated cells (Figure 6A). The expression of T-JAK2 and T-STAT3 which was localised mostly in the cytoplasm under the same experimental conditions remained unchanged (Figure 6B).

Paclitaxel-induced activation of JAK2 and downstream STAT3 were inhibited by CYT387, a potent small molecule JAK2 inhibitor (Figure 6A). Optimal inhibition of paclitaxel-induced JAK2/STAT3 activity was observed at 1 μM CYT387, which was subsequently used in all further experiments. The addition of CYT387 to paclitaxel-treated cells resulted in a significant reduction of P-STAT3 and P-JAK2 expression in HEY cells, compared to residual cells surviving paclitaxel only treatment (Figure 6A). However, the expression of total JAK2 and STAT3 expression remained unchanged in all treatment groups (Figure 6B).

### CYT387 inhibited paclitaxel-induced JAK2/STAT3 activation in ascites-derived tumor cells

**Figure 7:**
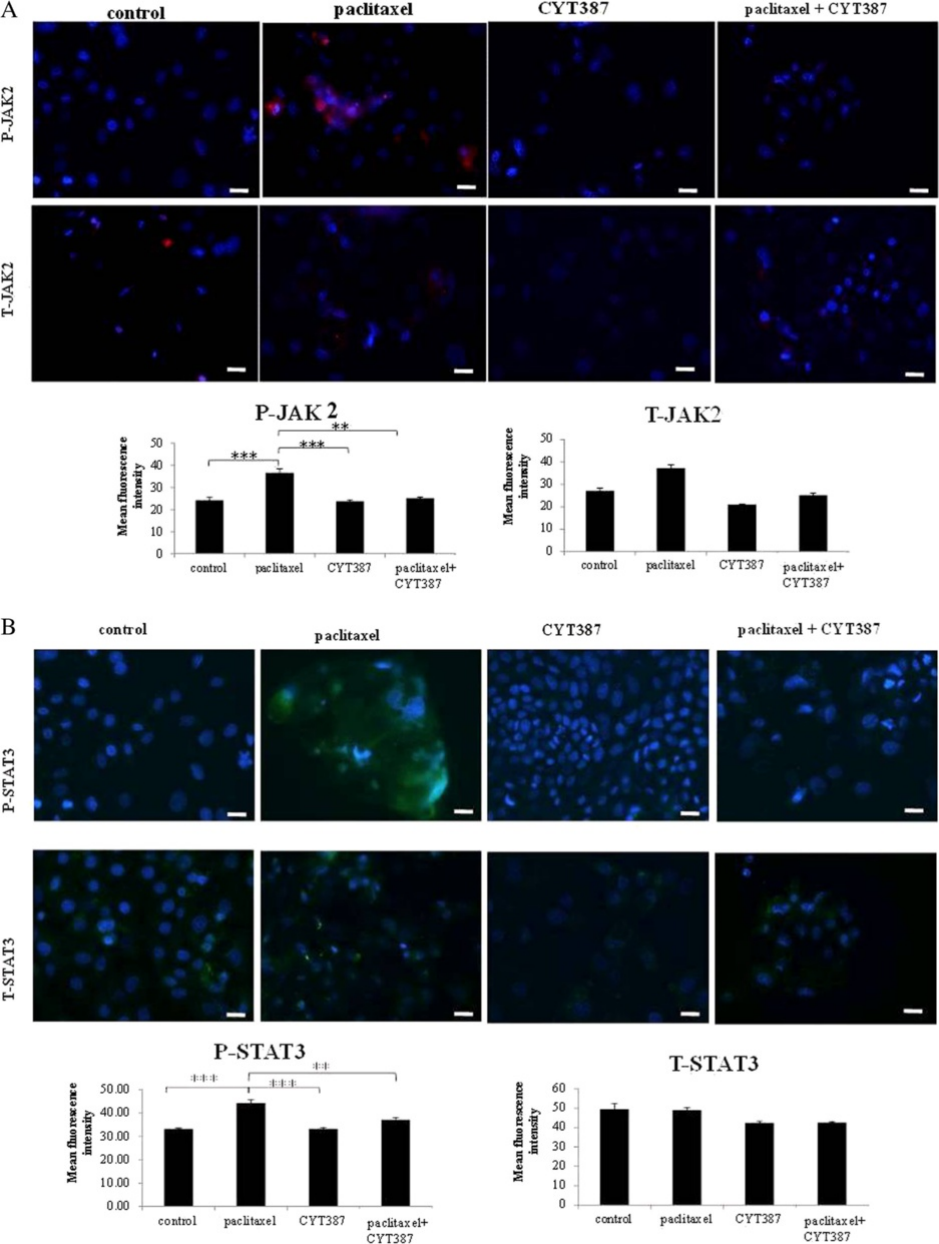
**Expression and localisation of phosphorylated and total JAK2 and STAT3 in control, paclitaxel and paclitaxel plus CYT387-treated ascites derived tumor cells. (A)** Expression and immunolocalisation of phospho (P)-JAK2 (Tyr-1007/1008) and total (T)-JAK2 in control, paclitaxel, CYT387 and combination of both treatments in ascites-derived tumor cells was evaluated as described in Figure 6A. Images are representative of three independent experiments performed in triplicate using three independent patient samples. Significant variations between the groups are indicated by **P<0.01, ***P < 0.001. **(B)** The expression of phospho (P)-STAT3 and total (T)-STAT3 was evaluated and quantified as described in Figure 6A. Significant variations between the groups are indicated by **P<0.01, ***P < 0.001. Magnification 200x; scale bar = 10 μM.

Consistent with HEY cell line, addition of CYT387 resulted in the inhibition of phosphorylation of JAK2 and STAT3 in paclitaxel-induced ascites-derived tumor cells (Ascites 9–11, Table 1), while the expression of T-JAK2 and T-STAT3 remained unchanged (Figure 7A and B).

### CYT387 treatment significantly reduced the CSC-like trait associated with paclitaxel treatment in HEY cells and ascites-derived tumor cells

**Figure 8:**
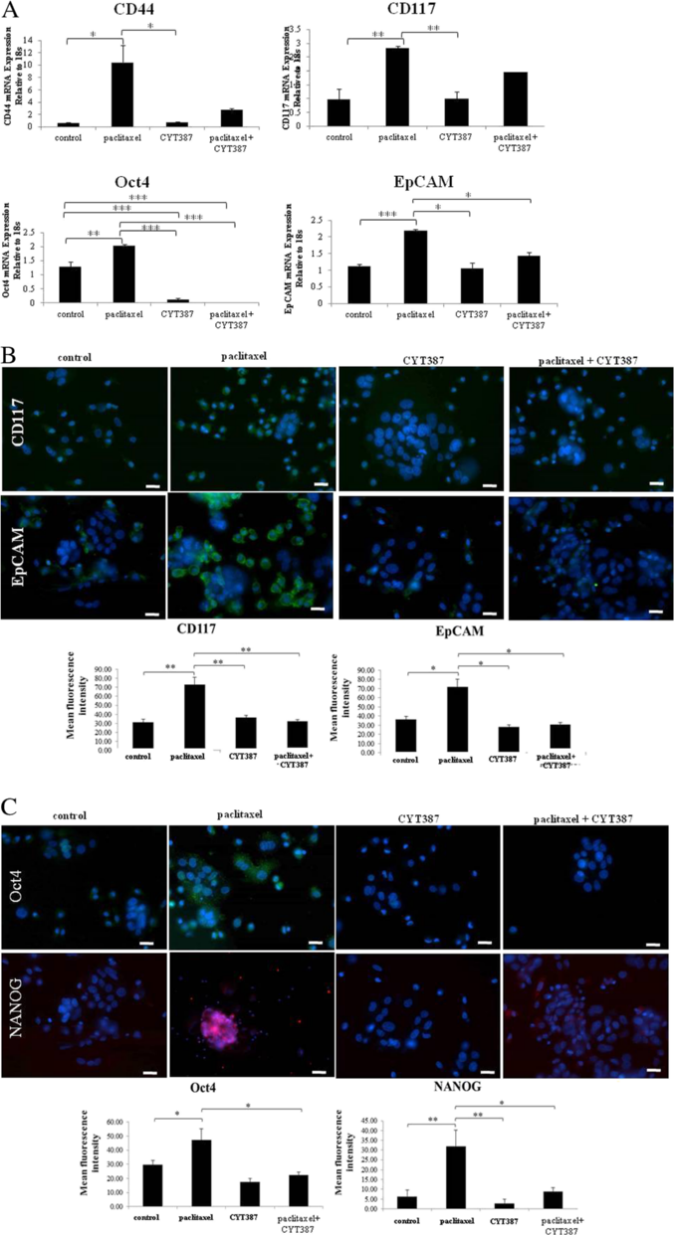
**Expression of CSC markers in control, paclitaxel, CYT387 and paclitaxel plus CYT387-treated HEY cells and ascites derived tumor cells. (A)** RNA from control and treated HEY cells was extracted, cDNA was prepared and qPCR for EpCAM, CD44, CD117 and Oct4 was performed as described in the Methods section. The resultant mRNA levels were normalized to 18S mRNA. The experiment was performed using four independent samples in triplicate. Significant intergroup variations are indicated by *P <0.05, **P<0.01, ***P < 0.001. **(B)** Expression and localisation of EpCAM, CD117 in ascites-derived tumor cells in response to paclitaxel, CYT387 and a combination of paclitaxel+CYT387 treatment was evaluated and quantified by immunofluorescence as described in Figure 1. Images are representative of three independent experiments using three independent patient ascites samples. Significant intergroup variations are indicated by *P <0.05, **P < 0.01. **(C)** The expression and localisation of embryonic stem cell markers NANOG and Oct4 in ascites-derived tumor cells was evaluated and quantified as described in Figure 1. Significant variations between the groups are indicated by *P <0.05, **P<0.01. Magnification 200x; scale bar = 10 μM.

We have previously shown the existence of CSC-like phenotypes in ovarian cancer cell lines, including the HEY cell line, primary and ascites-derived ovarian tumor cells isolated from ovarian cancer patients in response to cisplatin and paclitaxel treatments [14,32,45]. In order to assess if this phenomenon can be reversed by the inhibition of JAK2/STAT3 pathway by CYT387 in the presence of paclitaxel, we assessed the CSC-like profile of paclitaxel and CYT387-treated HEY cells at the mRNA level using qRT-PCR and compared that to control untreated as well as paclitaxel or CYT387 treatments alone (Figure 8A). Paclitaxel-treated HEY cells displayed significantly enhanced mRNA expression of CSC markers CD44, CD117, EpCAM and the embryonic stem cells markers Oct4 compared to control untreated or CYT387-treated cells (Figure 8A). However, this enhancement of CSC-like marker profile in response to paclitaxel treatment was abolished with the addition of CYT387, resulting in a significant reduction in the mRNA levels of Oct4 and EpCAM, while the mRNA expression of CD117 and CD44 was decreased but it was not significant (Figure 8A).

Similar to the results obtained with the HEY cell line, paclitaxel treatment of ascites derived tumor cells (Ascites 13–15) resulted in the significant enhancement of all tested CSC markers compared to their matched counterparts that did not receive paclitaxel treatment (Figure 8B-C). Treatment with only CYT387 did not result in any change in the expression of the CSC markers compared to the matched control counterparts (Figure 8B-C). However, the addition of CYT387 with paclitaxel to ascites-derived tumor cells demonstrated significant down regulation of CSC and embryonic stem cell markers when compared to the matched counterparts surviving paclitaxel only treatment (Figure 8B-C).

### The addition of CYT387 significantly enhanced the sensitivity of HEY cells and ascites-derived tumor cells to paclitaxel treatment

**Figure 9:**
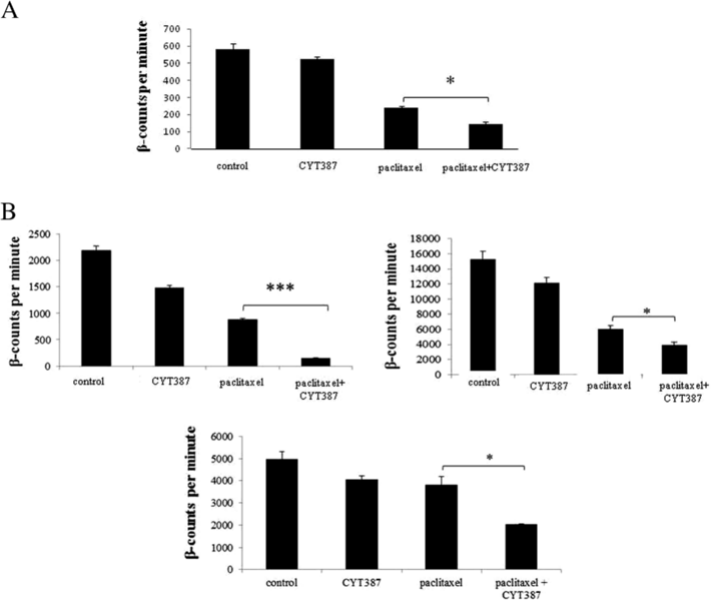
**Effect of CYT387 on the proliferation of HEY cells and ascites-derived tumor cells. (A)** HEY cells were treated with paclitaxel, CYT387 and combination of CYT387 and paclitaxel for three days. [^3^H]-thymidine was added and the cells were harvested as described in the Materials. The data is a representation of three independent experiments performed in triplicate. Significant variations between the groups are indicated by *P <0.05. **(B)** Ascites-derived tumor cells obtained from three independent patients were treated as described in Figure 9**A**. [^3^H]-thymidine uptake assay was performed as described in the Materials. The data is a representation of three independent experiments performed in triplicate on three ascites samples. Significant variations between the groups are indicated by *P <0.05, ***P < 0.001.

The growth pattern of HEY cells and ascites derived tumor cells (n = 3) in the presence of paclitaxel, CYT387 or paclitaxel plus CYT387 was determined by ^3^[H]-thymidine uptake assay. The HEY cell line and ascites-derived tumor cells were treated with ~ GI50 concentration of paclitaxel (1 ng/ml for HEY cells and 4-6 ng/ml for ascites tumor cells) and 1 μM concentration of CYT387, to determine if the combination of paclitaxel and CYT387 had an effect on the proliferation of cells compared to that obtained with the paclitaxel treatment alone (Figure 9A). The addition of CYT387 (1 μM) in the presence of paclitaxel sensitized HEY cells to paclitaxel treatment by significantly reducing the proliferation of cells by a further ~40% compared to paclitaxel only treated cells (Figure 9A). Similarly, addition of CYT387 (1 μM) sensitised the isolated ascites-derived tumor cells to paclitaxel by significantly reducing cell proliferation by approximately ~50-90% further than that obtained by paclitaxel alone treatment (Ascites 13–15, Table 1) (Figure 9B). Even though the proliferation rate of the three tumor populations derived from three patients was significantly different, CYT387 was able to sensitise all three ascites-derived tumor populations to paclitaxel (Figure 9B).

### Combination of paclitaxel and CYT387 treatment of HEY cells generated lower tumor burden in mice compared to tumor burden derived from paclitaxel-treated cells

**Figure 10:**
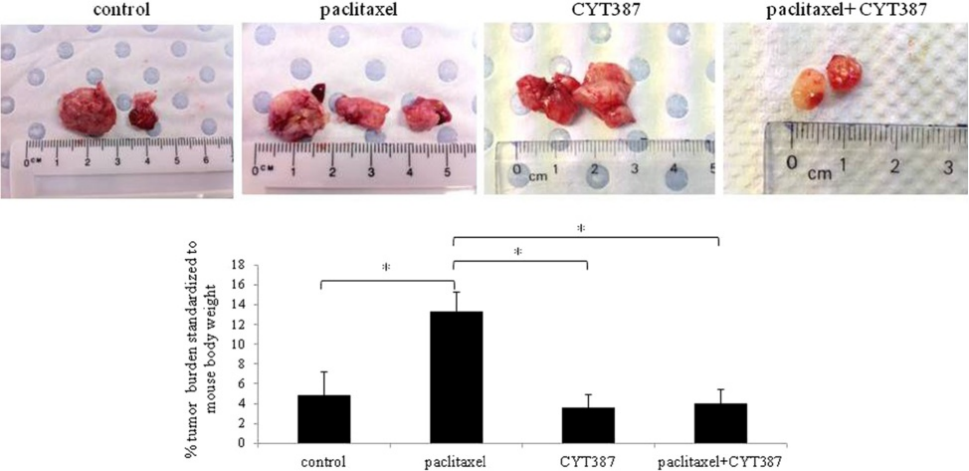
**Tumor burden in mice injected with control, paclitaxel, CYT387 and combination of paclitaxel plus CYT387-treated cells.** Total tumor burden obtained from mice 6 weeks after ip injection of control, paclitaxel-treated, paclitaxel plus CYT387-treated and combination of both CYT387 and paclitaxel-treated HEY cells (n = 5/group). 5×10^6^ cells were inoculated in each case. *P < 0.05, significant increase in tumor burden in paclitaxel-treated HEY cell derived tumors compared to control untreated group; and paclitaxel-treated HEY cell derived tumors to paclitaxel plus CYT387-treated cell derived tumors. Images represent tumors debulked from one mouse in each group.

The effect of the addition of CYT387 in conjunction with paclitaxel treatment was tested in *in vivo* mouse intraperitoneal (ip) HEY xenograft model used previously [45]. Mice (n = 5) injected with control untreated HEY cells developed solid tumors in the form of 3–4 small lesions (<0.5 cm^3^) in the peritoneum within six weeks. The average weight of the debulked tumors from the five control mice injected with untreated HEY cells weighed approximately 4.8% ± 2.3 of the total bodyweight (Figure 10). In contrast, mice injected with the same number (5×10^6^) of paclitaxel-surviving HEY cells produced a significantly larger tumor burden within the same time period, with the average tumors weighing ~ 13.32% ± 2 of the total body weight (Figure 10). On the other hand, tumors in mice injected with CYT387 plus paclitaxel treated cells weighed on average 4% ± 1.4 of the total mouse body weight (Figure 10). The average tumor weight in mice injected with CYT387 only treated HEY cells was ~ 3.5% ± 1.3 of the total body weight. In short, no significant difference in the tumor burden was observed between groups of mice injected with control untreated HEY cells or HEY cells treated with CYT387 (Figure 10). On the other hand, significantly lower tumor burden was observed in mice injected with HEY cells treated with a combination of paclitaxel and CYT387 versus mice injected with cells treated with paclitaxel alone (Figure 10). These results suggest that CYT387 in combination with paclitaxel reduces the tumor burden induced by paclitaxel only treatment, however, CYT387 on its own had no significant effect in reducing the tumor burden compared to control HEY cells-derived tumor burden.

### CYT387 in combination with paclitaxel significantly reduced CSC marker expression at the protein and mRNA levels in xenografts compared to xenografts derived from paclitaxel only treated cells

**Figure 11:**
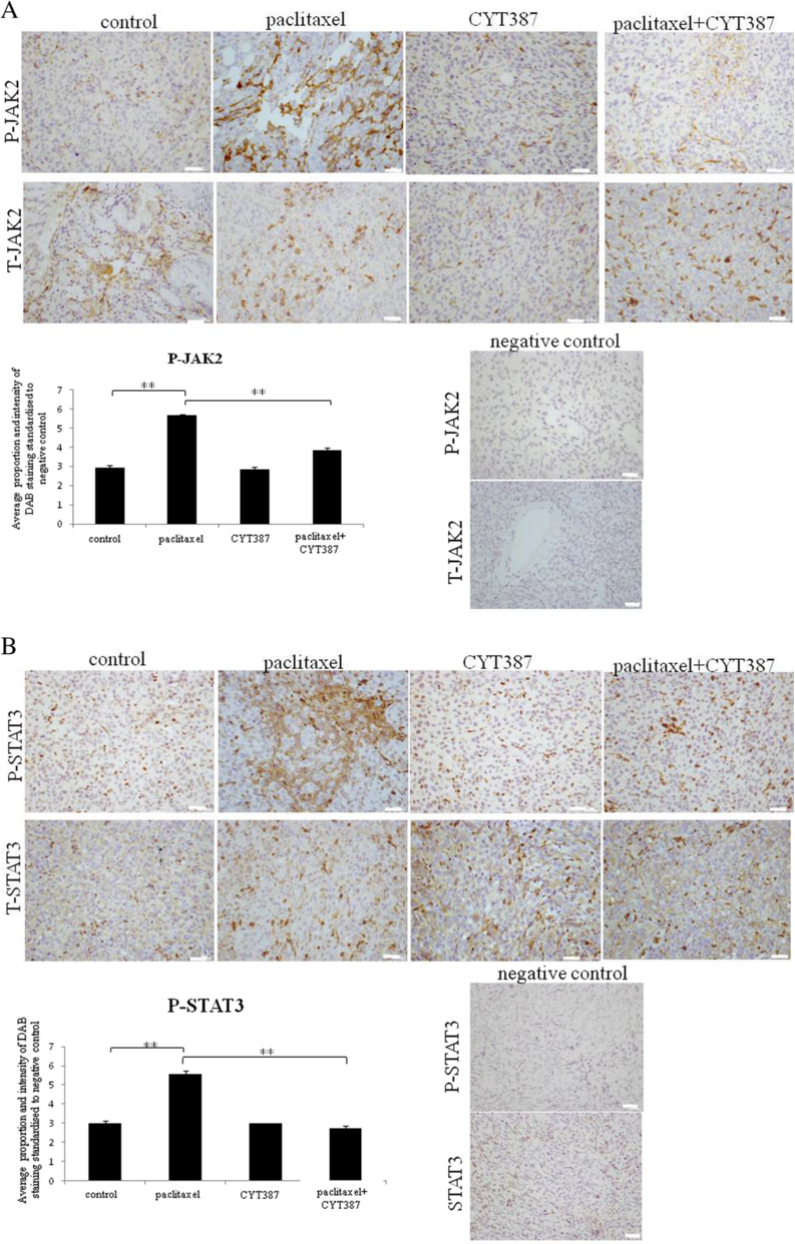
**Expression of P-JAK2, T-JAK2, P-STAT3 and T-STAT3 in mouse tumors generated from ip transplantation of control, paclitaxel, CYT387 and combination of paclitaxel plus CYT387- treated HEY cells. (A)** Immunohistochemistry staining of tumor sections for the expression of P-JAK2 and T-JAK2 was performed as described in Materials. Quantification of staining was obtained as described in Materials by using three independent xenografts. Significant variations between the groups are indicated by **P<0.01. **(B)** Tumor sections were stained for P-STAT3 and T-STAT3 and quantification of the data was obtained as described in Figure 11**A**. Significant variations between the groups are indicated by **P<0.01. Magnification 200×, scale bar = 10 μm

Debulked mouse tumors from mice inoculated with control, paclitaxel, CYT387 or paclitaxel plus CYT387 treated HEY cells were analysed using immunohistochemistry. Mouse tumors displayed positive staining for CK7 in all treatment-derived tumors cells (Additional file 2: Figure S2). In addition, positive staining for the proliferative marker Ki67 was also shown, with significantly reduced staining observed in tumors derived from paclitaxel plus CYT387 treatment surviving HEY cell-derived xenografts compared to paclitaxel only treated group (Additional file 2: Figure S2). We also performed immunohistochemistry analysis of the active (phosphorylated) and total JAK2 and STAT3 levels in mouse xenografts in the all four groups. Paclitaxel treatment derived tumors displayed significantly enhanced staining for both P-JAK2 and P-STAT3 compared to tumors derived from untreated or CYT387-treated HEY cells derived tumours (Figure 11A-B). On the other hand, tumors derived from HEY cells treated with paclitaxel plus CYT387 displayed significantly decreased staining for P-JAK2 and P-STAT3 compared to tumors derived from paclitaxel-treated cells, but expressed P-JAK2 and P-STAT3 at the same level as tumors derived from control untreated or CYT387-treated HEY cells (Figure 11A-B). The expression of T-JAK2 and T-STAT3 remained unchanged in control and treatment groups.

**Figure 12:**
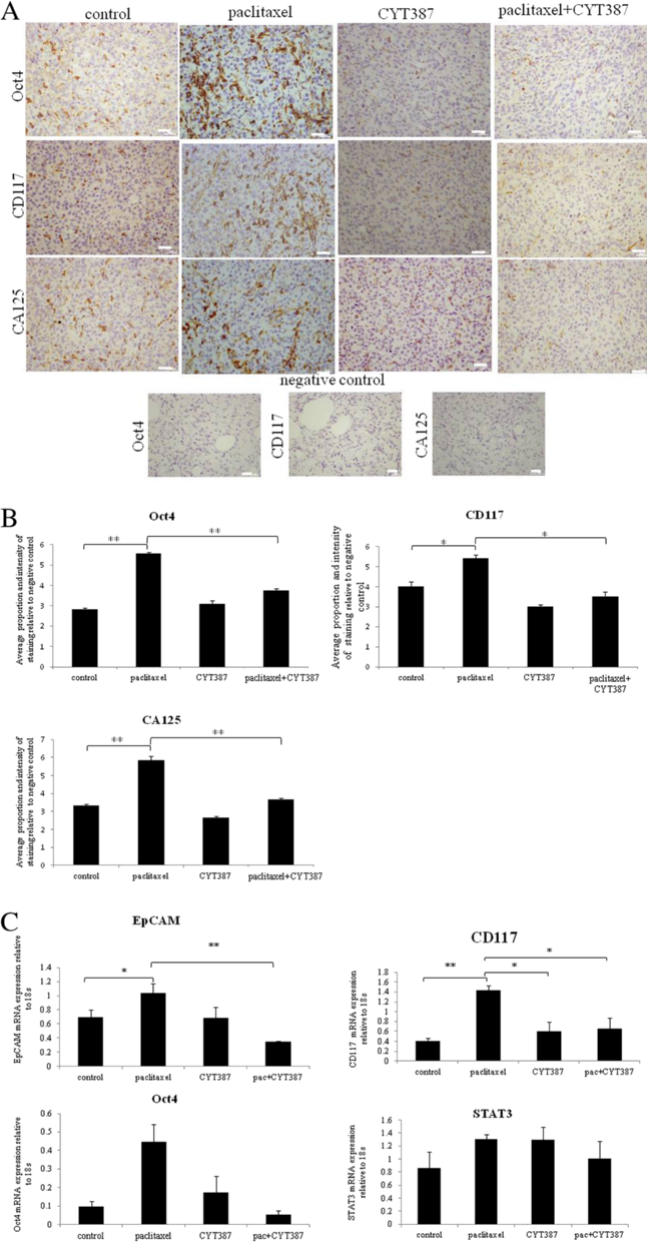
**Expression of CSC markers and CA125 in mouse tumors generated from ip transplantation of control, paclitaxel, CYT387 and combination of paclitaxel and CYT387-treated HEY cells. (A)** Immunohistochemistry staining of tumor sections for the expression of Oct4, CD117 and CA125 was performed as described in Figure 11A. **(B)** Quantification of Oct4, CD117 and CA125 staining was obtained as described in Figure 11A. Significant variations between the groups are indicated by *P<0.05 and **P<0.01. Magnification 200×, scale bar = 10 μm. **(C)** The mRNA expression of EpCAM, CD44, CD117 and Oct4 in control, paclitaxel, CYT387 and paclitaxel plus CYT387-treated HEY cells-derived xenografts was performed by q-PCR as described in the Methods section. The resultant mRNA levels were normalised to 18S mRNA. The experiments were performed using four independent samples in triplicate. Significant intergroup variations are indicated by *P <0.05, **P<0.01.

Coinciding with the activation of the JAK2/STAT3 pathway, immunohistochemistry analysis of mouse tumors for the CSC marker CD117 (c-kit), the embryonic stem cell marker Oct4 and the ovarian cancer marker CA125 revealed significantly enhanced staining in xenografts derived from cells surviving paclitaxel treatment compared to control untreated cells (Figure 12A-B). The expression of CD117, Oct4 and CA125 were reduced significantly in CYT387 plus paclitaxel treated cells-derived xenografts compared to paclitaxel only treated cells-derived xenografts, and was more comparable to xenografts derived from control untreated or CYT387-treated cells xenografts (Figure 12A-B). To determine if the changes in CSC markers seen in mouse xenografts derived from paclitaxel-treated and paclitaxel plus CYT387-treated HEY cells were consistent at the mRNA level; q-PCR was performed on cDNA prepared from RNA extracted from these tumors. Compared to xenografts derived from control untreated HEY cells, tumors derived from paclitaxel surviving cells showed significant enhancement of mRNA expression of CD117 and EpCAM (Figure 12C). Although paclitaxel treatment derived tumors showed significant enhancement of Oct4 at the protein level, and an apparent increase at the mRNA level, this enhancement was not statistically significant. An assessment of total STAT3 expression as the mRNA level showed no difference in STAT3 between all treatment groups.

### CYT387 in combination with paclitaxel does not reduce tumor invasion in mice

The tumor infiltration pattern within the peritoneal cavity in response to paclitaxel, CYT387, paclitaxel plus CYT387 was assessed using the H&E staining. In line with our previous study [45], sections of mouse organs (pancreas, liver, intestine, colon, and kidney) displayed infiltrating tumors with epithelial morphology. Although the addition of CYT387 with paclitaxel resulted in the significant reduction of tumor burden, an observation using a minimum of three mice in all treatment groups revealed no difference in the invasion pattern in all treatment regimens (images for pancreas and liver presented in Additional file 3: Figure S3.

## Discussion and conclusions

Despite advances in cancer treatment, a diagnosis of ovarian cancer is associated with a five year survival period of only 27% [3]. This is mainly due to the escape of a certain population of cells from the cytotoxic effect of therapies during treatment. These residual cells repopulate after a short duration (6–20 months), resulting in an aggressive recurrent tumor which consequently leads to patient’s mortality [2]. We have recently demonstrated that a short-term single treatment of ovarian cancer cells with cisplatin or paclitaxel resulted in residual cells with CSC-like trait capable of generating a significantly greater tumor burden in mice than control untreated cells [45]. In the current study, we demonstrate similar CSC-like trait in *in vitro* paclitaxel treated isolated ascites derived tumor cells with concomitant activation of the JAK2/STAT3 pathway. Using the HEY cell line model *in vivo,* we demonstrate suppression of paclitaxel treatment-induced tumor burden in mice by CYT387 a potent JAK2-specific inhibitor. This effect of CYT387 was mediated by substantially suppressing the phosphorylation of STAT3 at Tyr-705 *in vitro* and *in vivo*. This proof of principle study is the first to report that targeting the activated STAT3 induced by chemotherapy *in vitro* not only results in the abrogation of CSCs *in vitro* but also *in vivo,* and that this correlates with the reduction of tumor burden in mice. These data are novel and significant as the association between the JAK2/STAT3 pathway, ovarian CSCs and tumor burden in *in vivo* mouse models has not been demonstrated before.

After the first description of stem cells in ovarian tumors nearly eight years ago [24], significant progress has been made towards identifying, characterizing and understanding CSCs and their role in ovarian cancer [32,47,48]. However, despite these advances, ovarian cancer patients are still faced with incurable chemoresistant disease that may be attributed to a population of CSC-like cells [34]. In this study, using tumor cells isolated from the ascites of recurrent ovarian cancer patients we demonstrate that the emergence of a CSC-like phenotype in response to a short-term paclitaxel treatment *in vitro* coincided with enhanced staining of β-tubulin III and ERCC1, indicative of an acquired resistance to chemotherapy [49,50]. Enhanced expression of β-tubulin isotype III and/or ERCC1 are known to be expressed in tumor samples resistant to platinum and/or taxane-based therapies [49-51]. In addition, samples from advanced-stage ovarian cancer patients who developed clinical paclitaxel resistance showed increases in several β-tubulin subtypes including β-tubulin subtype III [10]. The fact that the ascites derived tumor cells used in this study not only showed significant enhancement in the expression of β-tubulin isotype III but also significant increase in the expression of ERCC1 in response to paclitaxel treatment, suggests that the cells surviving paclitaxel treatment may also had an elevated DNA repair mechanisms (genotoxic stress). This phenomenon may have been attributed to enhanced endogenous ERCC1 levels from previous exposure to carboplatin *in vivo* (in patients) before *in vitro* paclitaxel treatment of the isolated tumor cells (Table 1, Ascites 1–3).

An analysis of known CSC markers (CD117 and EpCAM) and embryonic stem cell markers (Oct4 and NANOG) at the protein and mRNA levels in seven ascites-derived tumor samples revealed enhanced expression of all tested CSC markers in response to *in vitro* paclitaxel treatment. This increase of CSC and embryonic stem cell markers in response to a short-term paclitaxel treatment shown in this study mirrors the response of HEY and OVCA 433 cell lines to paclitaxel treatment described previously [45]. The enhanced expression of a CSC-like phenotype in ascites-derived tumor cells coincided with the activation of the JAK2/STAT3 pathway.

In this study we also demonstrate a similar enhanced activation of the JAK2/STAT3 pathway in HEY cells within 2–3 days in response to paclitaxel treatment. Various cytokines and growth factors, including the gp130 family of cytokines that includes IL-6 and G-CSF, have been shown to activate the JAK2/STAT3 pathway [52]. Activated JAK2 auto-phosphorylates its receptors, and additionally phosphorylates STAT3, which results in the dimerization and translocation of STAT3 into the nucleus, where it binds to specific regulatory sequences to activate or repress transcription of target genes [37]. Recent studies have shown an acute drug-induced secretory response in tumor cells [53,54]. This results in the autocrine secretion of cytokines, which acts in favour of the tumor cells, and has a negative impact on the therapeutic response in patients [55,56]. We have also recently demonstrated enhanced autocrine secretion of IL-6 and G-CSF by HEY cells in response to paclitaxel-treatment *in vitro*[47]. This suggests that autocrine effects of IL-6 and G-CSF may activate the JAK2/STAT3 pathway in response to paclitaxel-treatment in HEY cells. The enhanced activation of JAK2/STAT3 may be required for the enhancement of CSC-like characteristics. This is evidenced by the suppression of JAK2/STAT3 activation and significantly suppressed expression of CSC markers at the mRNA level *in vitro* after the addition of CYT387 with paclitaxel to the HEY cell line. These effects of CYT387 resulted in the inhibition of proliferation of paclitaxel-treated residual ascites-derived tumor and HEY cells by a further ~40-90%.

The link between activation of the JAK2/STAT3 pathway and CSCs has been shown in a previous study on ovarian cancer, where the stem cell marker CD44 coupled with the embryonic stem cell marker NANOG have been linked with the activation of STAT3 in ovarian cancer cells [57]. Such activation of STAT3 in these cancer cells resulted in the expression of multidrug resistant genes and concomitant chemoresistance. These studies are consistent with reports demonstrating the STAT3 pathway to be a requisite for the proliferation and maintenance of glioblastoma stem cells [58], as well as rapidly cycling intestinal stem cells [59]. In addition, LIF and IL-6 mediated STAT3 dependent regulation of the Oct4-NANOG circuitry has been shown to be necessary to maintain the pluripotent inner cell mass, the source of embryonic stem cells [60]. These studies suggest a close relationship between the cytokine-mediated activation of the JAK2/STAT3 pathway and the survival of normal, cancer and embryonic stem cells. The activation of the JAK2/STAT3 pathway by paclitaxel in the current study may facilitate resistance to apoptotic pressures in paclitaxel-surviving cells, thus pushing the residuals cells into adopting a chemoresistant phenotype. This property of the activation of JAK2/STAT3 pathway in response to chemotherapy is not unique to the HEY cell line but has also been observed in tumor cells isolated from the ascites of ovarian cancer patients and OVCA 433 ovarian cancer cell line [13].

We and others have previously shown constitutive activation of STAT3 in high-grade ovarian carcinomas and suppression of the growth of ovarian cancer cells by inhibition of constitutive STAT3 activity [36,38]. Tyrosine phosphorylation of STAT3 has been considered to be more important than serine phosphorylation for the activation of STAT3 under oncogenic conditions [61]. Recent studies have demonstrated apoptosis, anchorage independent death and potentiating effects of chemotherapy response in ovarian cancer cells by inhibiting constitutively active STAT3 pathway [62]. This is consistent with a recent gene expression analysis of matched ovarian tumors and peritoneal metastasis which identified enrichment of genes of the JAK/STAT pathway in peritoneal metastasis [63]. However, ours is the first study that demonstrates that chemotherapy can induce early activation of the JAK2/STAT3 pathway above the levels normally present in cancer cells. As we show in this study, this phenomenon is crucial for the survival of chemotherapy-resistant CSCs which potentially are the drivers of repopulation and the eventual recurrent disease.

The novelty of the present study is the demonstration of the suppression of enhanced CSC-like characteristics observed in ovarian cancer cells after a single dose of paclitaxel treatment by CYT387 *in vitro*, and the retention of these characteristics in *in vivo* mouse xenografts. Tumor cells within the xenografts generated from paclitaxel and CYT387-treated cells had a lower proliferative potential as evaluated by low Ki67 staining, and a smaller tumor burden within the same time frame as that of the tumors derived from paclitaxel-treated cells. In addition, tumors derived from CYT387 and paclitaxel-derived cancer cells had a lower expression of CA125. Elevated level of CA125 is the hallmark of ovarian cancer diagnosis and frequently observed in recurrent disease [1,3]. CA125 expression has been shown to regulate the growth, tumorigenesis and metastasis of ovarian cancer cells as knock down of CA125 (deleted N-terminal region) completely abrogated the subcutaneous tumor forming ability of SKOV3 cells in nude mice [64]. Conversely, the same study showed that ectopic expression of CA125 with intact cytoplasmic tail enhanced ovarian tumor growth and metastases in SCID mice and the increased invasiveness of these cells *in vivo* correlated with classical EMT phenomenon (decreased expression of E-cadherin and increased expression of N-cadherin and vimentin) of the transfected cells *in vitro*. These findings provide evidence that CA125 plays a critical role in ovarian cancer cell growth, tumorigenesis and metastases. The relatively lower abundance of proliferative and tumorigenic markers in tumors derived from HEY cells treated with CYT387 and paclitaxel compared to paclitaxel-treated cells derived tumors suggest a crucial role of the JAK2/STAT3 pathway in maintaining chemotherapy-induced CSC phenotype in ovarian cancer. The fact that these characteristics induced by paclitaxel and CYT387 can be translated from *in vitro* to *in vivo* mouse xenografts suggest that these phenotypes once embedded in cancer cells becomes an intrinsic phenotype of the tumor cells and can be retained *in vivo.*

No significant differences in the invasion pattern were observed between control untreated, paclitaxel-treated, CYT387-treated and paclitaxel plus CYT387-treated cells-derived xenografts. This may be due to the small number of tumor xenografts analyzed or the fact that the cells only received a single short-term dose of paclitaxel and/or CYT387 treatments before inoculation into mice. Such short-term treatments even if induced invasiveness (if any) *in vitro* was not sustained during the six week tumor development in *in vivo* microenvironment. In future studies, this aspect of the work will be further investigated by systemic administration of paclitaxel and/or CYT387 in mice intraperitoneally inoculated with ovarian cancer cells. This is likely to show differences in the invasion pattern imposed by paclitaxel and/or CYT387 in vivo.

**Figure 13:**
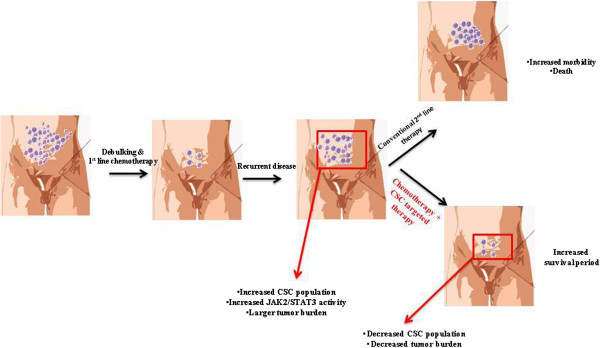
**A model of chemoresistance and associated recurrence in ovarian cancer.** Adapted from Googleimages, Wisegeek.com. At diagnosis, majority of the ovarian cancer patients present with high-grade tumors and associated ascites which contains tumor cells as well as CSCs. After the first line of ‘traditional chemotherapy’ treatment majority of the tumor cells are eradicated leaving behind residual tumors which mainly consist of chemoresistant CSCs with enhanced level of phosphorylated JAK2/STAT3. These patients are in remission for 6–22 months. At recurrence, patients present with larger tumor burden which has increased numbers of CSCs. Under the current treatment protocol most patients are treated with subsequent lines of chemotherapy (which differs in patients), resulting in successive recurrences which ultimately leads to patient mortality. However, if the patients are treated with ‘traditional chemotherapy’ in combination with JAK2/STAT3 inhibitors, this will eradicate CSCs during the first line of treatment, and/or subsequent lines of treatments. This consequently may result in decreased tumor burden with increased disease free survival period and better treatment outcomes.

Repopulation of ovarian cancer cells that escape the cytotoxic effects of first line of chemotherapy has an unknown mechanism. This is particularly important as ovarian cancer patients who do not receive treatment for a short period (6–22 months) while in remission after surgery and first line of chemotherapy [4]. During this time it is hypothesized that the residual tumor cells may have ample time to adapt to the changed microenvironment, repopulate and progress the disease to a symptomatic state. Generation of CSC-like characteristics in response to cytotoxic pressures may be one of the important pathways by which chemonaive ovarian cancer cells escape the cytotoxic effects of first line chemotherapy, adapt to the changed abdominal microenvironment and repopulate over time to develop a recurrent disease [47]. Our results firmly establish that the taxane chemotherapy treatment activates the JAK2/STAT3 pathway in ovarian cancer cells and isolated tumor cells from ascites. We have also demonstrated that CYT387 not only inhibits JAK2/STAT3 signaling but also the expression of paclitaxel-induced CSCs that subsequently results in reduced tumor burden in mice. Our results provide strong evidence that CYT387 potentiates these effects not only *in vitro* but also *in vivo*. Taken together, the findings from this study lend support to further investigation into the use of CYT387 in combination with chemotherapy (paclitaxel) for the better management of ovarian cancer patients. Prospective events demonstrating the activation of JAK2/STAT3 pathway and the involvement of residual CSC-like cells in response to chemotherapy treatment, which are the ultimate source of recurrent disease, are depicted in Figure 13.

## Competing interest

The authors declare that they have no competing interest.

## Author’s contribution

KA designed the study, performed the experiments and contributed to the writing of the manuscript, RL and HZ helped with the animal experiments, OM and MQ, provided the human samples and edited the manuscript, CB, provided reagents and was involved with the discussion of the manuscript, EWT and JKF edited the manuscript, NA conceived the idea, designed the study and contributed to the writing of the manuscript. All authors read and approved the final manuscript.

## Pre-publication history

The pre-publication history for this paper can be accessed here:

http://www.biomedcentral.com/1471-2407/14/317/prepub

## Supplementary Material

Additional file 1: Figure S1 mRNA expression of CSC markers in control and paclitaxel treated ascites-derived tumor cells. RNA from the control and matching paclitaxel treated ascites-derived tumour cells was extracted cDNA was prepared and q-PCR for EpCAM, NANOG CD44, CD117, Oct4, JAGGED, STAT3 and E-cadherin was performed as described in the Methods. The resultant mRNA levels were normalized to 18S mRNA. The experiments were performed using five independent patient samples; the resulting mRNA results were then pooled for analysis. Significant variation is indicated by *P < 0.05. Click here for file

Additional file 2: Figure S2 **(A-B)**: Immunohistochemistry expression of Ki67, cytokeratin 7 (CK7) in mouse tumors generated from ip transplantation of control, paclitaxel, CYT387 and combination of CYT387 and paclitaxel-treated HEY cells. **(A)** Tumor sections were stained and scoring for the staining of Ki67 and CK7 was performed as described in Figure 11. Magnification 200X, scale bar = 10 μm. **(B)** Significant variations between the groups is indicated by **P < 0.01. Click here for file

Additional file 3: Figure S3 H and E staining of control and treated HEY cell derived-tumor associated infiltrated organs in mice. 5 × 10^6^ cells were injected ip in each mouse. Histological images of liver and pancreas showing infiltration of control, paclitaxel-treated, CYT387 and combination of paclitaxel and CYT387-treated HEY cells. Arrows indicate tumor cells invading the respective organs. Magnification 200×, scale bar = 10 μm. Click here for file
