# Supplementary figures and images for: Inhibition of the JAK2/STAT3 pathway in ovarian cancer results in the loss of cancer stem cell-like characteristics and a reduced tumor burden
Source: BMC Cancer. 2014 May 6;14:317. doi: 10.1186/1471-2407-14-317 (PMC4025194; doi:10.1186/1471-2407-14-317)

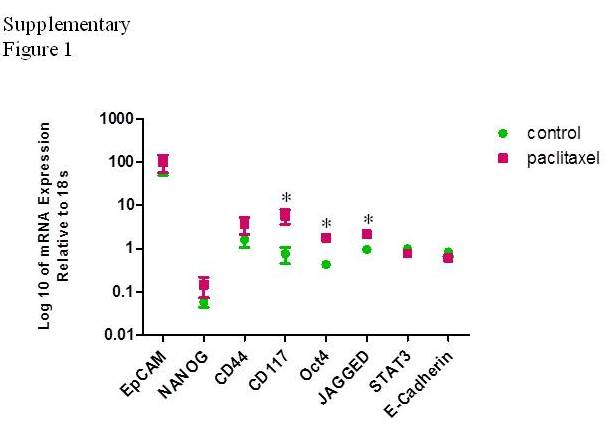

Supplement: Additional file 1: Figure S1 — mRNA expression of CSC markers in control and paclitaxel treated ascites-derived tumor cells. RNA from the control and matching paclitaxel treated ascites-derived tumour cells was extracted cDNA was prepared and q-PCR for EpCAM, NANOG CD44, CD117, Oct4, JAGGED, STAT3 and E-cadherin was performed as described in the Methods. The resultant mRNA levels were normalized to 18S mRNA. The experiments were performed using five independent patient samples; the resulting mRNA results were then pooled for analysis. Significant variation is indicated by *P < 0.05. [file 1471-2407-14-317-S1.jpeg]

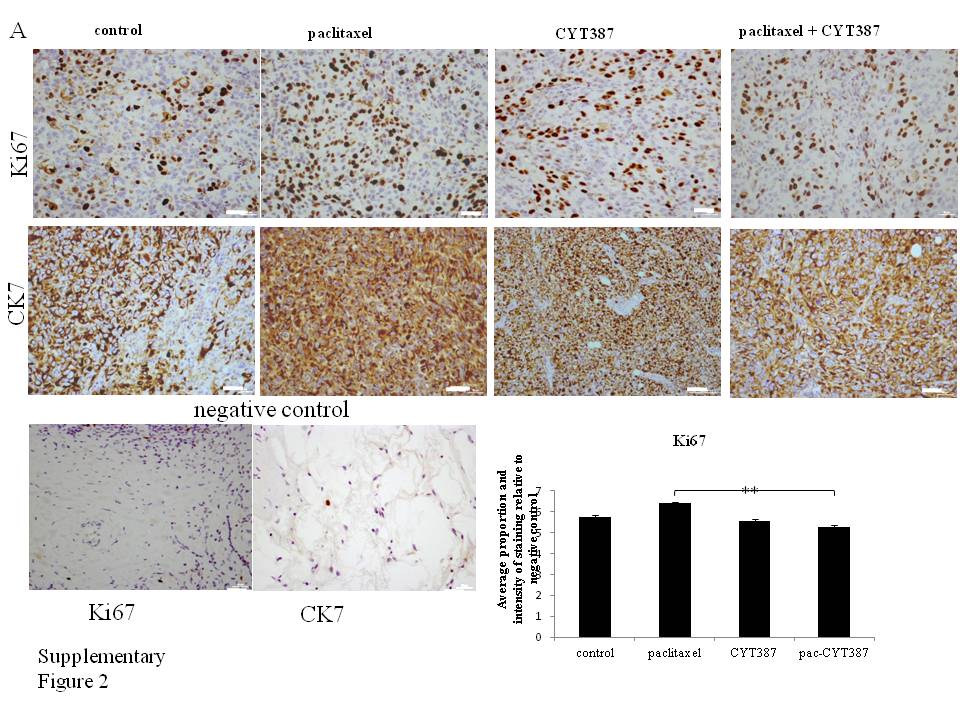

Supplement: Additional file 2: Figure S2 — (A-B): Immunohistochemistry expression of Ki67, cytokeratin 7 (CK7) in mouse tumors generated from ip transplantation of control, paclitaxel, CYT387 and combination of CYT387 and paclitaxel-treated HEY cells. (A) Tumor sections were stained and scoring for the staining of Ki67 and CK7 was performed as described in Figure 11. Magnification 200X, scale bar = 10 μm. (B) Significant variations between the groups is indicated by **P < 0.01. [file 1471-2407-14-317-S2.jpeg]

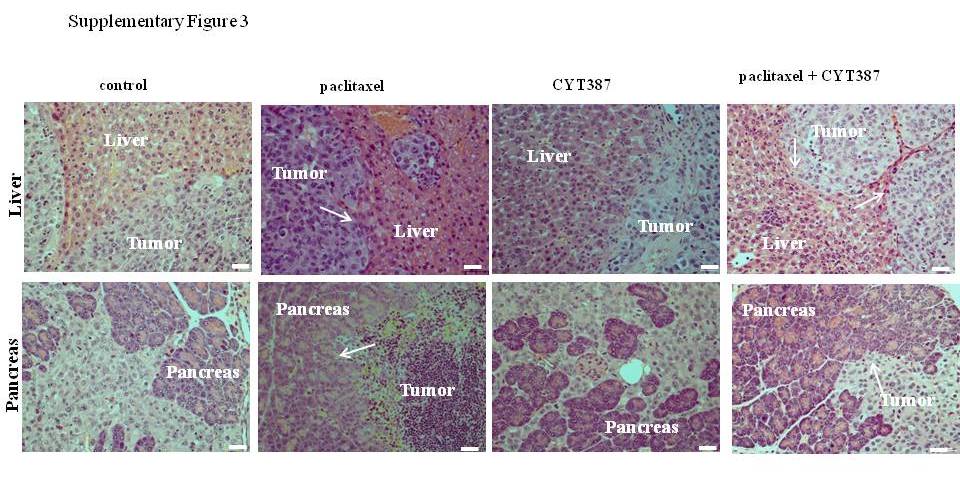

Supplement: Additional file 3: Figure S3 — H and E staining of control and treated HEY cell derived-tumor associated infiltrated organs in mice. 5 × 10^6^ cells were injected ip in each mouse. Histological images of liver and pancreas showing infiltration of control, paclitaxel-treated, CYT387 and combination of paclitaxel and CYT387-treated HEY cells. Arrows indicate tumor cells invading the respective organs. Magnification 200×, scale bar = 10 μm. [file 1471-2407-14-317-S3.jpeg]
